# Health commodities inventory management performance and associated challenges in selected public health facilities of South Gondar Zone, Amhara Region, North Central Ethiopia: A mixed-methods cross-sectional study

**DOI:** 10.1371/journal.pone.0349202

**Published:** 2026-05-15

**Authors:** Zewdu Tessema, Tesfaye Tsigu, Zelalem Tilahun, Gashew Tessema, Ayenew Berhan, Eskinder Eshetu Ali

**Affiliations:** 1 Ethiopia Red Cross Society, Debre Tabor Branch, Debre Tabor, Ethiopia; 2 Global Health and Supply Chain Specialist UNICEF, Addis Ababa, Ethiopia; 3 Department of Social and Administrative Pharmacy, School of Pharmacy, College of Medicine and Health Science, Addis Ababa University, Addis Ababa‌‌, Ethiopia; 4 Sport Science Academy, Bahir Dar University, Bahir Dar, Ethiopia; 5 Department of Medical Laboratory Science, College of Health Sciences, Debre Tabor University, Debre Tabor, Ethiopia; Mutah University, JORDAN

## Abstract

**Introduction:**

In the healthcare system, the supply chain refers to the coordinated flow of physical and technical resources needed to deliver quality patient care while adhering to budget constraints. To achieve this, measuring inventory management performance is essential. This process involves evaluating the efficiency and effectiveness of activities against predefined standards using established indicators. Therefore, this study aimed to assess the inventory management performance of health facilities in the South Gondar Zone, Northcentral Ethiopia.

**Methods:**

This study employed a facility-based cross-sectional study design with a concurrent mixed-methods approach. Eighteen health facilities, representing 30% of the 57 health facilities in the South Gondar Zone, were included in this study. Quantitative data were collected using structured tools and analyzed in Microsoft Excel. Meanwhile, qualitative data were gathered from expert interviews and thematically analyzed to identify the major challenges faced by these facilities.

**Results:**

Emergency ordering was frequent, with 83.3% of facilities placing at least one emergency order in 2020–2021, increasing to 100% placing two or more orders in 2021–2022. Inventory record accuracy was suboptimal, with an average accuracy rate of 71.3% (±9.6), and only 16.7% of facilities maintained up-to-date stock cards. While reporting rates were high (98.2%), report completeness (52.8%) and accuracy (72.2%) remained inconsistent. Storage performance was inadequate, as only 16.7% of facilities met acceptable storage standards (≥80% of criteria), despite high compliance with vaccine cold-chain requirements (94.1%). Qualitative findings identified human resource constraints, limited training, weak data management systems, and inadequate infrastructure as key contributors to poor inventory performance.

**Conclusion:**

The performance of inventory management for health commodities in the study health facilities is low. This is primarily due to a shortage of skilled personnel, insufficient management support and budget, a lack of ongoing professional training, poor record-keeping practices, inaccurate data quality, and inadequate storage conditions. This investigation discovered that the frequency of emergency orders is the most important indicator, and all responsible bodies should pay more attention to reducing it, in addition to implementing other sound inventory management operations to build a strong health care delivery system.

## Introduction

### Background

In health care settings, a reliable supply of health commodities, such as medicines, laboratory reagents, chemicals, and consumable medical supplies, is essential for the detection and treatment of diseases [1]. An effective inventory management system is crucial to the supply chain because it ensures that health commodities are readily available to meet consumer demand when needed [2]. This system can help reduce healthcare costs while maintaining high-quality service for patients. Additionally, it enhances the efficiency and throughput of the healthcare system [3].

As defined by the American Production and Inventory Control Society (APICS), Supply Chain Management (SCM) is the comprehensive, strategic coordination of the entire product lifecycle—from raw material sourcing and procurement through production, distribution, and final delivery to the customer. Inventory management, by contrast, is a narrower, operational component within SCM that focuses specifically on controlling stock levels, reducing carrying costs, and ensuring products are available when and where they are needed. In healthcare facilities, the primary goal is to reduce costs while maintaining the quality of patient care. This is achieved by enhancing the efficiency and throughput of the healthcare system [4]. Effective inventory management of health supplies is essential for healthcare delivery. To avoid frequent stockouts of health commodities and other inventory management challenges, particularly in public health facilities, it is crucial to implement robust inventory management systems alongside other supply chain management practices [5]. In line with this perspective, a five-year hospital-based study emphasized the importance of proactive supply chain strategies, strengthened supplier coordination, and enhanced system preparedness to mitigate drug shortages and ensure continuity of care [5].

A study on inventory management performance for laboratory supplies in public hospitals within the Jimma zone of Southwest Ethiopia revealed that at least one or two emergency orders were placed within six months in four hospitals. Key bottlenecks for inventory management identified in this study were budget constraints, a lack of timely administrative support, insufficient personnel commitment, and periodic shortages of commodities from suppliers [6].

A facility-based descriptive cross-sectional quantitative method, alongside a qualitative approach conducted in the West Wollega Zone of Ethiopia to examine the performance of inventory management for family planning, maternal, and child health medicines in public health facilities, revealed that the average medicine availability was 61.3%, and the mean stock-out duration was 70.71 days. Out of the medications assessed, 78.4% had bin cards, but only 52.45% (374) of these bin cards were accurate. The report submission rate was 84.06%, with 40.52% of the reports and resupply forms submitted on time. Additionally,62.93% of these forms were complete, and 59.48% were accurate. Due to a lack of robust inventory management techniques, vital medicines have been reduced by 30%, and medical delivery lead times have increased by 10% to 20%. In the second scenario, inventory errors cut profits by 10%, while misplaced products cut profits by 25%. This demonstrates how performance monitoring can reveal a problem and lead to better inventory management [7]. The study also identified several concerns related to inventory management, including issues with suppliers, a lack of human resources, administrative challenges, and insufficient computer equipment [8].

Despite the government's strong support and adequate budget allocation for enhancing pharmaceutical supply chain management, there are still concerns regarding the effective implementation of the inventory control system [9]. In addition, supply chain management is recognized as a vital competitive tool, there has been limited research on the assessment of supply chain operational excellence in managing health commodities inventory in Ethiopia. This suggests a significant knowledge gap regarding the performance of supply chain management practices in the country. Therefore, this study aimed to quantitatively assess key inventory management performance indicators, including inventory accuracy, stock out rate, reporting quality, and storage compliance, and to qualitatively explore system-level factors influencing performance in selected public health facilities of South Gondar Zone, Ethiopia, between 2019/2020 and 2020/2021. It highlights the importance of regularly monitoring key indicators and establishing effective feedback mechanisms. The research also provides an empirical overview of current inventory management performance for health commodities. It offers baseline data to monitor future changes and improvements in selected public health institutions in the South Gondar Zone, North Central, Ethiopia.

## Methods and materials

### Study area, study design, and period

From January 5 to March 30, 2022, a health institution-based study using a concurrent mixed-methods design that combined both quantitative and qualitative approaches was conducted. The quantitative approach used a structured questionnaire to collect data from the primary data source to assess inventory management performance using various standard operating procedures and key performance indicators with different perspectives, whereas the qualitative approach employed a semi-structured questionnaire to capture information that cannot be gathered quantitatively and to supplement the quantitative data. The study took place in government health facilities located in the South Gondar Zone, Amhara Region, North Central Ethiopia. This zone consists of 18 districts and has an estimated population of 2.36 million people [9]. According to the South Gondar Zonal Health Department report of 2020/2021, the health facilities in the area include 10 public hospitals, 96 health centers, and 406 health posts.

### Source and study population‌‌

The source population included all public health facilities in South Gondar Zone, while the study population comprised hospitals and health centers providing Antiretroviral therapy (ART) services. These health-care facilities serve a large number of people and have a complex inventory management system. In addition, ART medication is one of the tracer health commodities chosen for this investigation to show inventory management performance comprehensively. In total, 8 hospitals and 49 health centers offered ART and Prevention of Mother-To-Child Transmission (PMTCT) services, and only facilities operational before 2019 were included in the study. Data were collected through review of RRFs, bin cards, stock cards, Model 19 (receiving vouchers), Model 22 (issuing vouchers), purchase orders, and the Health Commodity Management Information System (HCMIS). Storage conditions were assessed through direct observation. Health professionals working in these facilities served as the source population, with only those having at least six months of experience included in the study population.

### Eligibility criteria

#### Inclusion criteria.

The study focused on health-care facilities that are providing ART and PMTCT services and were established before 2019, and health professionals working in these facilities for at least six months were included in the study population.

#### Exclusion criteria.

The study excludes health facilities damaged by recent conflicts and health facilities that are being prepared as a COVID-19 treatment center.

### Sample size determination and sampling techniques

The required number of health facilities to be included in the survey was determined based on Nthambi, 2014; [10], which states that the sample size of the study should be 30 percent of the target population in proportion to the size of the stratum, as this has been proven to be sufficient for a descriptive survey study. Of the 64 health facilities providing ART and PMTCT services in South Gondar Zone, six were excluded due to local conflicts, and one was designated as a COVID-19 treatment center, leaving 57 eligible health facilities. From these, 30% (18) health facilities were selected using stratified random sampling by level of care: one referral hospital, two primary hospitals, and fifteen health centers [11,12].

Based on the 2020/2021 top ten diseases report of South Gondar Zone and consultations with logistics experts from the Zonal Health Department, Amhara Regional Health Bureau, and Bahir Dar Ethiopian Pharmaceutical Supply Agency Hub, a total of 25 health commodities were selected for the study. The selection process followed the Management Science for Health Inventory Management Assessment Tool (IMAT) and included first-line medicines for the leading morbidities, consistent with the national standard treatment guideline

Health facilities are required to provide six times per year to corresponding pharmaceutical suppliers, or every two months, all RRF reports from the selected 18 health facilities were analyzed for RRF data quality and reporting rate.

Key informants for the qualitative data included store managers, pharmacy heads, health center directors, and hospital chief executive officers, as they possess greater knowledge on the topic. The sample size was kept manageable to ensure detailed data collection within available time and resources [13].

### Data collection procedure

Quantitative data were collected through observation checklists for storage standards, physical stock counts, and review of prescriptions and relevant records. Use of bin cards and RRFs was assessed from July 8, 2019, to July 7, 2021. This data was collected from 5/1/2022–30/3/2022. Whereas, qualitative data were gathered from key informants using an Amharic language interview that lasted at least 30 minutes, recorded, and later translated into English by the principal investigator [14].

### Data quality control and analysis

Before the commencement of the study, a training was given for data collectors about the objective of the study and the data collection tool and procedure. In addition, a pretest was performed before the actual data collection. Data were collected by trained pharmacy-background data collectors under the daily supervision of the principal investigator. The quantitative data were analyzed using Microsoft Excel version **13**. Excel was utilized for data entry, cleaning, coding, and analysis. Descriptive statistical techniques—including frequencies, percentages, means, and standard deviations—were computed using its built-in functions and data analysis tools. Additionally, tables and charts were generated within Excel to facilitate clear presentation and interpretation of the findings, while qualitative data trustworthiness was ensured through clear participant briefing, audio recordings, and peer review. Qualitative data does not have the same level of validity and reliability control as quantitative data. As a result, participants were very clear about the nature and purpose of the research, and audiotaped recordings and peer examinations were used in order to establish trustworthiness and improve the validity and reliability of the qualitative part of the research.

The research was carried out in groups, with descriptive statistics being used (frequency, mean, Standard deviation, and percentage). The quantitative analysis was intentionally limited to descriptive statistics to align with the primary objective and design of the study. This study was fundamentally diagnostic and exploratory, aiming to assess the current status of health commodities inventory management and identify operational gaps rather than to test hypotheses or establish causal relationships (inferential statistical analysis). The following formula measures were used.

1. Stock out rate$\left(\frac{\text{The number of facilities that experienced a stockout of a specific product}}{\text{Total number of facilities that are expected to offer that product}}\right)\times \text{100}$
2. Percent of stores that met standard criteria$\left(\frac{\text{Number of stores that fulfilled the criteria}}{\text{Total number of stores visited}}\right)\times \text{100}$
3. Inventory accuracy rate$\left(\frac{Number\;of\;Stock\;record\;count=Physical\;stock\;count}{Total\;number\;of\;items\;assessed}\right)\times 100$
4. RRF data quality
  - RRFs accuracy rate=$\left(\frac{\text{Number of accurate reports }}{\text{Total number of RRF reports submitted}.\;}\right)\times \text{100}$
  - RRF timelines rate=$\left(\frac{\text{Number of times reached the report}.}{\text{Total number of reports submitted}}\right)\times \text{100}$
  - RRF completeness rate$\left(\frac{\text{Number of completed reports}}{\text{Total number of reports submitted}}\right)\times \text{100}$
  - RRF reporting rate =$\left(\frac{\text{The number of facilities submitted their report on the specified schedule}}{\text{The total number of facilities expected to submit their report}}\right)\times \text{100}$
5. Percentage of availability and usage status of IPLS and/or APTS tools =$\left(\frac{\text{Number and usage of tools present}}{\text{Total number of IPLS Tools selected }}\right)\times \text{100}$

For qualitative data, all key informant responses were entered and evaluated in Microsoft Word, which was used to help with data coding and theme analysis. Framework analysis was used to explain a theme or concept and included definitions of words and related concepts. It is helpful when a researcher is attempting to compare themes or concepts [15].

We attentively listened to the audio and read the transcriptions, details, and field notes after the interview to review the collected data and analyze it using the six processes outlined below.

1. **Familiarize with the data:** We understood the obtained data and spent time going through it and becoming acquainted with the various themes in the data.
2. **Generating initial codes**: During this phase, we began to categorize the data and identify concepts.
3. **Searching for themes or sub-themes:** During this step, the researcher generated themes and identified links between themes.
4. **Reviewing themes:** The themes identified in the third phase are evaluated and analyzed at this point. We also compared the results to other studies that used a similar approach. Even though studies differ in many ways, this comparison aids in validating data analysis results.
5. **Defining and naming themes:** This phase entails naming and defining all of the themes. We also looked at whether any of the topics could be combined or removed from the analysis.
6. **Producing the report:** The final stage is the study's actual authoring. We produced a summary paragraph and detailed descriptions for each of the themes.

The significance of the idea, probable concept connections, and compliance with the research purpose were all examined throughout the analytical processes.

### Operational definitions

- Stock out rate: The drug is termed a stock-out if the ending balance on the logistical recording tool (bin card) is zero in the six months before data collection, and there is no usable quantity in physical stock during survey time.
- RRF data quality: It is the timeliness, accuracy, and completeness of logistical data.
- The completeness of reports: A report is complete if all of the columns for each product listed in the RRF report are filled out, unless the facility does not manage the product, and all columns of the RRF are completed, reporting period, and facility name are recorded [16].
- Timeliness: A report is considered timely if health facilities submit their RRF to the higher supplier EPSS-Ethiopian pharmaceutical supply service (EPSS) until the 10^th^ day and to the woreda health office until the 5^th^ day after the reporting period [17]).
- RRF's data accuracy: When transferring data from the bin card to RRF, if there is no discrepancy, and there are no calculation mistakes in each column for the selected health commodities, RRF is regarded as correct [18].

### Ethics approval and consent to participate

Ethical approval letter for this study was obtained from the School of Pharmacy Ethical Review Committee, Addis Ababa University, with reference number Ref No: ERB/SOP/390/14/2021, and a permission letter to conduct the study was obtained from each health institute, and a written consent to participate in the study was obtained from each study participant.

## Results

### Quantitative findings

The study assessed Integrated Pharmaceutical Logistics System (IPLS) and Auditable Pharmaceutical Transactions and Services (APTS) tool availability and utilization, frequency of emergency orders, storage practices, inventory accuracy, RRF data quality, and reporting rates in 18 selected health facilities. Storage practices were observed during the survey, while retrospective data were collected to assess the percentage of availability, usage status of IPLS and/or APTS tools, and the total percentage average for two years, from July 8, 2019, to July 7, 2021.

Despite some challenges regarding the consistent usage of bin cards and Internal facility reports and requestion form (IFRR), the availability of various IPLS and APTS resources such as bin cards, RRF, IFRR, new Model-19 (Receiving voucher), and new Model-22 (issuing voucher), tablet counting trays, calculators, and scissors is met in almost all facilities evaluated. Stock cards are available in almost 66.67% of the health facilities, and 16.7% health facilities have an up-to-date stock card (**Table 1**).

**Table 1:** Percentage of IPLS and/ APTS tools/resources availability and use status in selected public health facilities of South Gondar Zone, Northcentral, Ethiopia,2022(n = 18).

S. N	Tools/Resources	Availability		Use status	
		Yes N (%)	No N (%)	Yes N (%)	No N (%)
1	New Model-19	18(100)	0(0)	18(100)	0(0)
2	New Model-22	18(100)	0(0)	18(100)	0(0)
3	Bin card	18(100)	0(0)	18(100)	0(0)
4	Stock card	12(66.67)	6(33.33)	3(16.67)	15(83.33)
5	RRF®	18(100)	0(0)	18(100)	0(0)
6	IFRR^¥^	18(100)	0(0)	16(88.89)	2(11.11)
7	E-recording	10(55.56	8(44.44)	10(55.56)	8(44.44)
8	Dispensing registers	16(88.89	2(11.11)	12(66.67)	6(33.33)
9	Labeling stickers	10(55.56)	8(44.44)	7(38.89)	11(61.1)
10	Tablet counting trays	17(94.44)	1(5.56)	17(94.44)	1(5.56)
11	Calculators	18(100)	0(0)	18(100)	0(0)
12	Scissors	18(100)	0(0)	18(100)	0(0)
13	STG*	18(100)	0(0)	0(0)	18(100)
14	Formulary manual	17(94.44)	1(5.56)	17(94.44)	1(5.56)
15	Facility-specific list	12(66.67)	6(33.33)	10(55.56)	8(44.44)
	**Total average**	88.15%	11.85%	74.07%	25.93%

*STG-Standard treatment guideline; ^¥^IFRR-Internal facility reports and requestion form; ^®^RRF-facility reports and requestion form

**Fig 1:**
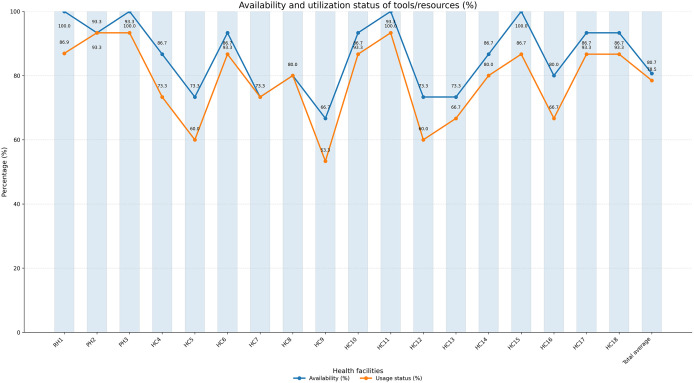
Percentage of IPLS and / APTS tools or resources availability and usage status in selected public health facilities of South Gondar Zone, Amhara, Northcentral, Ethiopia, 2022 (n = 18). RH-referral hospital; PH-primary hospital; HC-health center.

*The average IPLS and/or APTS tools/resources availability and usage status by the selected health facilities were 86.65% ± 11.23 (Minimum = 66.67, Maximum = 100) and 78.48% ± 12.62 (Minimum = 53.33, Maximum = 93.33), respectively* **(Fig 1).**

### Frequency of emergency orders

**Fig 2:**
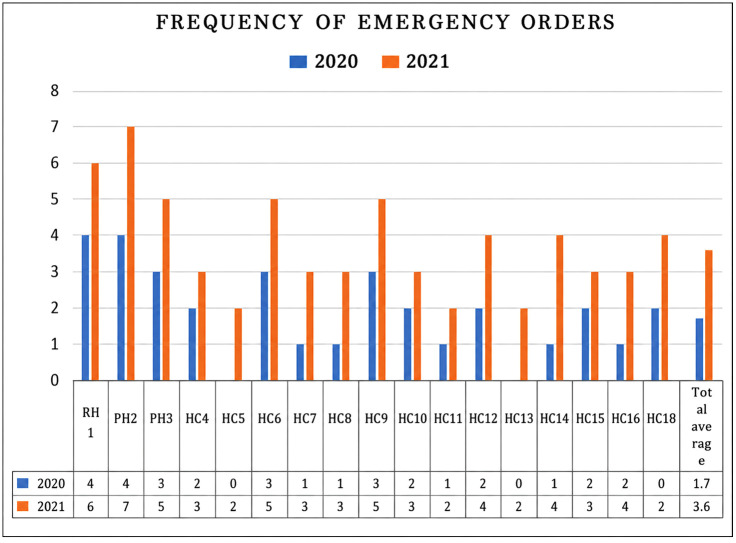
Frequency of emergency orders in selected public health facilities in South Gondar Zone, North Central, Ethiopia, 2022 (n = 18). RH-referral hospital; PH-primary hospital; HC-health center.

IPLS recommends placing emergency orders when stock falls below the defined threshold, which is two weeks for hospitals and health centers. Key informant interviews revealed that, in addition to using the RRF, 83.3% health facilities placed at least one emergency order during 2020–2021. By 2021–2022, due to frequent stock-outs, all health facilities had placed at least two emergency orders for program health commodities (**Fig 2**).

### Percentage of inventory accuracy and stock-out rate

Data quality was assessed by comparing bin card balances with physical counts and checking whether bin cards were properly used and updated. A bin card was considered accurate if its balance matched the physical count. None of the health facilities met the recommended inventory accuracy rate of 100%; the average accuracy was 71.33% ± 9.63 (range: 52–92%). Over the previous six months, each facility experienced stock-outs of an average of 15.28% of the selected commodities (**Table 2**).

**Table 2:** Percentage of inventory accuracy and stock out rate in selected health facilities of South Gondar Zone, Northcentral, Ethiopia,2022(n = 18).

Health facilities name (Code)	Inventory accuracy rate at survey time (%)	Stock out rate during survey time n (%)	Stock out rate during the past six months, n (%)
RH1	64	2(8%)	12(48%)
PH2	80	4(16%)	16(64%)
PH3	92	6(24%)	19(76%)
HC4	68	3(12%)	13(52%)
HC5	76	4(16%)	16(64%)
HC6	72	3(12%)	12(48%)
HC7	72	8(32%)	17(68%)
HC8	52	10(40%)	19(76%)
HC9	60	6(24%)	18(72%)
HC10	68	7(28%)	12(48%)
HC11	76	9(36%)	18(72%)
HC12	72	7(28%)	11(44%)
HC13	84	5(20%)	17(68%)
HC14	76	3(12%)	14(56%)
HC15	60	8(32%)	15(60%)
HC16	64	3(12%)	17(68%)
HC17	80	4(16%)	16(64%)
HC18	68	5(20%)	13(52%)
**Total average**	71.33	5.39(21.28%)	15.28(61.11%)

RH-referral hospital; PH-primary hospital; HC-health center.

### RRF data quality and reporting rate

Reliable recordkeeping is essential for the proper execution of inventory management in order to offer a regular flow of health commodities to our clients, and RRF must then be communicated to higher levels for effective logistics decision-making. The study gathered all RRF submitted by chosen health facilities from proximal pharmaceutical suppliers, Ethiopian Pharmaceutical Supply Service (EPSS), Bahir Dar, and Gondar branches between 2019/2020 and 2020/2021. The majority of health facilities submit the RRF report, although maintaining the schedule, completeness, and accuracy is a challenge. The study health facilities submitted 98.15% (212) of the planned 216 RRF reports between 2019/2020 and 2020/2021 (**Table 3**).

**Table 3:** Percentage of RRF data quality and reporting rate in selected public health facilities of South Gondar Zone, North central, Ethiopia, 2022(n = 18).

Health facilities name (Code)	2019/2020				2020/2021			
	Reporting rate N (%)	Timelines N (%)	Completeness N (%)	Accuracy N (%)	Reporting rate N (%)	Timelines N (%)	Completeness N (%)	Accuracy N (%)
RH1	6(100%)	5(83.33%)	2(33.33%)	4(66.67%)	6(100%)	4(66.67%)	3(50%)	3(50%)
PH2	6(100%)	3(50%)	2(33.33%)	3(50%)	5(83.33%)	4(80%)	4(80%)	5(100%)
PH3	6(100%)	6(100%)	5(83.33%)	6(100%)	6(100%)	5(83.33%)	6(100%)	6(100%)
HC4	5(83.33%)	4(80%)	2(40%)	3(60%)	6(100%)	6(100%)	1(16.7%)	3(50%)
HC5	6(100%)	3(50%)	2(33.33%)	2(33.33%)	6(100%)	5(83.33%)	4(66.67%)	2(33.33%)
HC6	6(100%)	6(100%)	3(50%)	2(33.33%)	6(100%)	6(100%)	5(83.33%)	3(50%)
HC7	5(83.33%)	3(60%)	1(20%)	2(40%)	6(100%)	6(100%)	4(66.67%)	3(50%)
HC8	6(100%)	6(100%)	0(0%)	3(50%)	6(100%)	5(83.33%)	2(33.33%)	2(33.33%)
HC9	6(100%)	6(100%)	6(100%)	5(83.33%)	6(100%)	6(100%)	6(100%)	6(100%)
HC10	6(100%)	5(83.33%)	5(83.33%)	4(66.67%)	6(100%)	6(100%)	3(50%)	5(83.33%)
HC11	6(100%)	6(100%)	4(66.67%)	6(100%)	6(100%)	5(83.33%)	5(83.33%)	5(83.33%
HC12	6(100%)	1(16.67)	2(33.33%)	5(83.33%)	6(100%)	5(83.33%)	5(83.33%)	6(100%)
HC13	6(100%)	5(83.33%)	3(50%)	4(66.67%)	6(100%)	6(100%)	5(83.33%)	6(100%)
HC14	6(100%)	6(100%)	4(66.67%)	4(66.67%)	6(100%)	4(66.67%)	6(100%)	6(100%)
HC15	6(100%)	5(83.33)	2(33.33%)	6(100%)	6(100%)	6(100%)	3(50%)	5(83.33%)
HC16	6(100%)	2(33.33%)	6(100%)	5(83.33%)	6(100%)	3(50%)	5(83.33%)	5(83.33%)
HC17	5(83.33%)	4(80%)	4(80)	5(100%)	6(100%)	5(83.33%)	3(50%)	6(100%)
HC18	6(100%)	6(100%)	3(50%)	4(66.67%)	6(100%)	4(6667%)	5(83.33%)	6(100%)
**Total average**	97.22%	75.93%	36.11%	67.60%	99.07%	84.25%	69.44%	76.85%

RH-referral hospital; PH-primary hospital; HC-health center.

### Percentage of health facilities that met standard storage practice

The majority of the health facilities,94.12%, used proper vaccine storage temperatures (2–8°c) and followed first-in-first-out inventory management processes. On the other hand, only 23.53% of the health facilities have fire safety equipment such as a functional fire extinguisher (**Table 4**).

**Table 4:** Percentage of storage standard observation criteria in selected public health facilities of South Gondar Zone, Northcentral, Ethiopia, 2022(n = 18).

S.N	Observation criteria	Frequency
		Yes (%)
1	Storage sites are securely locked.	12(70.59)
2	Access to storage and pharmacy is limited to authorized personnel only	6(35.29)
3	The storeroom is clean, all trash removed, sturdy shelves, and organized boxes	5(29.41)
4	Cold storage facilities for vaccines are maintained at (2–8 degrees centigrade)	16(94.12)
5	Inventory is protected from excessive humidity according to product specifications	13(76.47)
6	Inventory is protected from harmful light sources (Must not be in direct sunlight)	13(76.47)
7	Corrosive and combustible products are held separately	10(58.82)
8	Separate Storage of non-pharmaceutical products from drugs	7(41.18)
9	Shelves must be at least 10 cm off the floor, no more than 2.5m high, and 30 cm away from the walls.	6(35.29)
10	Fire safety equipment is available and accessible, e.g., Functional fire extinguisher	4(23.53)
11	Cartons placed with arrows up, labeled, and expiry dates visible	15(88.24)
12	Separate damaged or expired products and remove them from stock	15(88.24)
13	Sufficient space for free tracking of items	9(52.94)
14	Inventory is arranged in a manner accessible for first-to-expire, first out (FEFO)	16(94.12)
15	Packages and containers are closed	13(76.47)
16	Packages are clean in both the pharmacy and the store room	10(58.82)
17	Packages and boxes are not crushed	13(76.47)
	**Total average**	**63.32%**

**Fig 3:**
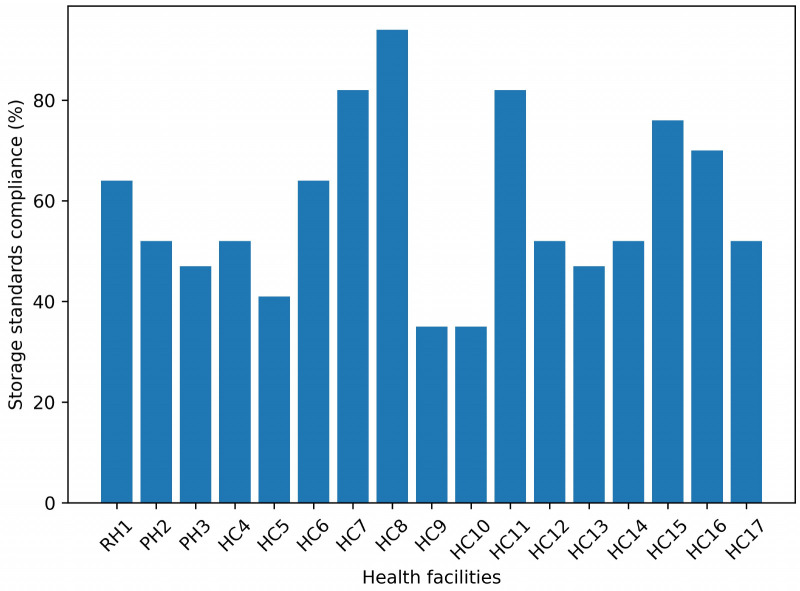
Percentage of storage standards by study health facilities in South Gondar Zone, Northcentral, Ethiopia, 2022(n = 18). RH-referral hospital; PH-primary hospital; HC-health center.

The percentage of health facilities meeting acceptable storage conditions by facility type was also assessed in this study. On average, only 16.7% of the health facilities met acceptable storage conditions (80% of the criteria or more) (**Fig 3**).

### Qualitative study findings

#### Challenges identified using key informant interviews.

To investigate the difficulties impacting inventory management performance that result in poor service delivery, interviews were performed with 21 key informants working in the selected health institutions, South Gondar Zonal Health Department, and EPSS Bahir Dar branch (**Table 5**).

**Table 5:** Socio-demographic characteristics of key informants in selected organizations and public health facilities of South Gondar Zone, Amhara, North central, Ethiopia,2022(n = 21).

Variables	Categories	Frequency n (%)
Sex	Female	5(23.8)
	Male	16(76.2)
Age in years	20-30	11(52.38)
	31-40	7(33.33)
	40-50	3(14.29)
Place of work	EPSS	<3(4.76)
	ZHD	<3(4.76)
	Hospital	6(28.57)
	Health center	13(61.9)
Educational level	Diploma	10(47.61)
	First degree	9(42.86)
	Master’s degree	2(9.52)
Work experience in years	1_5	9(42.86)
	6_10	12(57.14)
Position	Management	6(28.6)
	Pharmacy-related staff	15(71.4)

EPSS=Ethiopian pharmaceutical supply service; ZHD=Zonal health department.

Qualitative data were manually analyzed using an inductive thematic approach. The investigators reviewed audio recordings, took notes, and coded the data, which were then translated into English. Codes were organized to identify subthemes, which were merged, labeled, and described. Four main themes emerged, and the findings were presented narratively.

#### Logistics management information system data quality issues.

Fifteen percent of participants believed that employees’ uneven sense of ownership and commitment, resulting from limited internal career paths, a lack of integrated human resource utilization, and high staff turnover, are the primary issues in this sector, influencing the overall quality of inventory management data.

#### Issues with the capability of the health commodities storehouses.

Twenty-one percent of participants believed that storage room infrastructures were not standardized, such as having a very small ceiling, a dirty floor, a lack of proper shelves and pallets, and so on. Also, medications are not kept apart from supplies and equipment. Our storage practice requirements are also impacted by store manpower shortages.

One respondent mentioned that:

“*We had a serious problem with our store room, particularly its cleanliness, as a result of a lack of attention on the part of our cleaners; even when we contact them to clean the space, they refuse to answer. Furthermore, the building's specifications are deficient. The ceiling is quite low and cannot accommodate enough things, particularly when receiving.” [Male, druggist working at hospital]*

Another key informant added that:

“*To your surprise, our warehouse contains rodents such as rats that contaminate fluids such as normal saline, as well as ready-to-use therapeutic foods that increase our wastage and stockout rates. There is also an overabundance of waste commodities in our storeroom, which has overcrowded our storage area due to poor, timely disposal practices and a lack of coordination among health facilities to exchange near-expired products.” [Male, dru*gg*ist working at health center]*

#### Challenges for continuous professional development (CPD).

Ninety-five percent of participants believed that there is insufficient short and long-term training and supervision to fill employees’ skill gaps, which helps to improve inventory management performance. This is due to financial constraints and the responsible bodies’ lack of focus on the area. Some respondents also claimed that staff motivation and gap-filling activities do not exist. This could be because of recent events in our country, which have affected a variety of health-related activities, including short and long-term training. Because of our country's instability, numerous stakeholders have been diverted away from issues like employee empowerment.

One respondent also argued that:

*“…Since my first day of work at this facility, I haven't seen any training. I believe that no one considered us (pharmacy professionals) to be medical professionals. Even when some treatment guidelines are revised, we do not receive any information, which causes some friction between the pharmacy department and prescribers regarding prescription-related concerns.” [Male, pharmacist working at hospital]*

Another participant mentioned that:

*“Even though our facility asked us to propose an area where there is a skill gap that needs to be filled through onsite training and discussion, our department was unable to do so because onsite training does not provide incentives (financial support) for employees, which is a bad habit of requesting financial support to attend training all of the time, and it has an impact on our work performance.”* [*Female, pharmacist working at hospital]*

#### Administrative-related issues.

According to some participants, inequitable competent human resources and resource mobilization for proper workflow, as well as a lack of competence to apply regulatory frameworks such as external audits, have a negative influence on overall inventory management performance.

One key informant added that:

*“Lack of attention by non-professional government officials to public health facilities to allocate enough budget to run various supply chain activities successfully. The push approach used by EPSS and the regional health bureau for some health commodities has a significant effect on inventory management performance….” [Male, pharmacist working at hospital]*

Another respondent also mentioned:

“*We don't have any transport vehicles for health commodities transportation as a significant health institution that serves a huge number of customers, thus we try to employ private contract vehicles the majority of the time. This affects the product's quality and timeliness of delivery...*.*” [Male, druggist working at health center]*

## Discussion

The current study reflected that the average availability and usage status of tools in the selected health facilities for the deployment of a robust inventory management system were 86.65% and 78.48%, respectively. The value for the availability of assessed tools in this study is significantly lower than in the national survey of Ethiopian hospitals average of 90%, but slightly higher than or comparable to health centers, close to 80% [19]. The discrepancy could be attributed to the fact that the tools utilized to evaluate this indication were not identical to those employed in this investigation. The finding, however, is matched with the assessment report completed for APTS sites in Ethiopia,86.4% [20]. Appropriate health service delivery is the outcome of having accessible data and working tools. Moreover, it should be noted that the usage of those evaluated tools alone may not be adequate; the tools must also be updated on a regular basis and carefully documented.

In this study, 83.3% (15/18) of health facilities placed at least one emergency order during 2020–2021. By 2021–2022, 100% (18) health facilities had placed at least two emergency orders, mainly for program health commodities. This increase may be attributed to disruptions in the health supply chain and higher demand for health commodities due to the COVID-19 pandemic and internal population displacements. These findings are consistent with a national assessment in Ethiopia, which reported that 68% of hospitals and 43% of health centers placed at least one emergency order within three months [21]. Another study in Southwest Ethiopia also found that at least one or two emergency orders were placed within six months in four of the hospitals [5]. Stockout was caused by poor selection, quantification, procurement, and insufficient stock control and management, and unexpected service demand or increased patient flow. Each dimension of supply chain management difficulties has a detrimental impact on responsiveness, collaboration, flexibility, and cost performance. As a result, emergency orders were usually placed.

In terms of keeping accurate and timely records of stocks, which alerts logistics personnel about the facility's stock status and allows them to take corrective action, this study found that the average inventory accuracy rate in the study area is 71.33%, which is higher than the study 52.45% conducted in public health facilities of the West Wollega zone, Ethiopia [6]. This study's inventory accuracy rate value is likewise significantly greater than that conducted in East Shewa Zone, Ethiopia, 28.5% [21]. The difference could be attributed to the health products utilized and the store managers’ commitment to keeping bin cards updated regularly. The workload, a lack of appropriate human resources, and store managers’ dedication all have an impact on our operations in the logistical data management system, such as the inventory accuracy rate, according to the key informants in the current study's qualitative sections. The inventory accuracy rate or logistic recording accuracy is critical in the management of health commodities since it contributes to the production of trustworthy data for determining the proper quantity and type of commodities. When bin cards or logistic data are not updated, the flow of information is disrupted, resulting in overstocking or understocking and the expiration of medications.

The study assessed retrospectively the RRF reporting rate, timeliness, completeness, and accuracy in the selected health facilities from July 8, 2019, to July 7, 2021. The average performance was 98.13%, 80.09%, 52.78%, and 72.22%, respectively. These findings were lower than findings from public health facilities in the Oromia Special Zone, Ethiopia,100%, 86.2%, 86.2%, and 82.8%, respectively [22]. However, the value of these indicators for this study was higher than the study,84.06%, 40.52%, 62.93%, 59.48% as described above [6]. Particularly in the first year of the study, or 2019/2020 in this study area, RRF completeness was very low,36%, providing insufficient information to decision makers. The variation could be due to differences in the report feedback system and the competency of assigned professionals. Participants in key informant interviews were also told that a lack of well-functioning electronic software precluded them from sending appropriate transaction reports, such as RRF, to various stakeholders. Decent RRF data quality is crucial to assist in transmitting accurate information to the higher level every two months to enable suitable and effective decision making by giving reliable information on their prior performance and stock on hand using the same RRF form.

In terms of storage standards practice, only16.7% (3/18) of the selected health facilities met acceptable storage conditions (80% of the criteria or more), which is lower than the study done at public health facilities of Dire Dawa city administration in Ethiopia,50% [23]. The majority of the health facilities, 94.12% (16/18), used proper vaccine storage temperatures (2–8°c) and first-in-first-out inventory management processes. However, only 23.53% (4/16) of health facilities have a functional fire extinguisher. The total percentage of storage standards in the study area was around 59.82%, which is lower than the recommended 80% value but higher than the study (50%) found in South West Showa Zone, Ethiopia, 50% [24]. The discrepancy could be attributed to differences in research area, criteria employed, and management assistance.

## Conclusion

The study revealed substantial systemic weaknesses across multiple dimensions of health commodities inventory management. Notably, there were significant deficiencies in Report and Requisition Form (RRF) data quality, characterized by incomplete, inconsistent, and untimely reporting. Inventory records were frequently inaccurate, with discrepancies between recorded and physical stock levels, indicating poor data integrity and weak internal control mechanisms. In addition, storage practices did not meet standard guidelines, with issues such as improper organization, inadequate space utilization, and suboptimal environmental conditions that could compromise product quality and safety.

Overall, the performance of health commodities inventory management was found to be unsatisfactory and below acceptable standards. This poor performance was driven by a combination of interrelated factors. There was a clear shortage of adequately trained and skilled personnel, limiting the effective implementation of inventory management procedures. Management support was insufficient, particularly in terms of supervision, resource allocation, and strategic oversight, while budget constraints further restricted essential activities such as procurement, infrastructure improvement, and capacity building. Moreover, the absence of continuous professional training and mentorship programs contributed to persistent knowledge and skills gaps among staff. Record-keeping practices were weak and often manual, increasing the likelihood of errors and data loss. As a result, data used for decision-making were frequently inaccurate or outdated, undermining planning and forecasting processes. Poor storage conditions further exacerbated these challenges, increasing the risk of product damage, expiration, and wastage. These systemic issues collectively led to frequent stock-outs of essential health commodities, disrupting service delivery and negatively affecting patient care outcomes.

### Recommendations

Future research should adopt longitudinal and interventional designs to better assess changes in inventory performance over time and the impact of targeted system improvements. Expanding evaluation to include forecasting accuracy, procurement lead times, wastage rates, and digital system functionality would provide a more comprehensive understanding of supply chain performance. Broader geographical comparisons are also needed to enhance generalizability. Additionally, evaluating structured operational improvement frameworks within public health supply chains may be beneficial, as integrating lean efficiency principles with Six Sigma process control has been shown to improve process reliability and service quality (13).

### Limitations of the study

Several limitations should be considered when interpreting the findings of this study. The cross-sectional design restricts the ability to draw causal inferences between system-related challenges and inventory performance outcomes. Important supply chain performance indicators such as forecasting accuracy, procurement lead time, and wastage rates were not assessed, which limits the comprehensiveness of the evaluation. The study was conducted within a single geographical zone, which may limit the generalizability of the findings to other regions with different resource availability, infrastructure, and administrative contexts. In addition, reliance on retrospective document review introduces the possibility of documentation bias, as the accuracy of reported indicators depends on the completeness and reliability of facility records. The qualitative findings may also be influenced by recall bias and social desirability bias, despite efforts to ensure trustworthiness.

## Supporting information

S1 File Final data PLOS One. (XLSX)

## Abbreviations

APTS: Auditable Pharmaceutical Transactions and Services
EPSS: Ethiopian Pharmaceutical Supply Service
IPLS: Integrated Pharmaceutical Logistics System
RRF: Report and request form
